# Bioactive compounds and antioxidant activity of *Rosa canina L.* biotypes from spontaneous flora of Transylvania

**DOI:** 10.1186/1752-153X-7-73

**Published:** 2013-04-23

**Authors:** Ioana Roman, Andreea Stănilă, Sorin Stănilă

**Affiliations:** 1 Department of Land Measurements and Science, University of Agricultural Sciences and Veterinary Medicine, 3-5 Mănăştur str, Cluj-Napoca 400372, Romania; 2 Food Science and Technology Faculty, University of Agricultural Sciences and Veterinary Medicine, 3-5 Mănăştur str, Cluj-Napoca 400372, Romania; 3 Department of Technical Sciences and Soil Sciences, University of Agricultural Sciences and Veterinary Medicine, 3-5 Mănăştur str, Cluj-Napoca 400372, Romania

**Keywords:** Rosehip fruits, Ascorbic acid, Polyphenols, Flavonoids, Antioxidant activity, Natural antioxidants

## Abstract

**Background:**

The theoretical, but especially the practical values of identifying the biochemical compounds from the *Rosa canina L.* fruits are of present interest, this aspect being illustrated by the numerous researches. It was reported that the *Rosa canina L.* fruit, with its high ascorbic acid, phenolics and flavonoids contents, have antioxidant, antimutagenic and anticarcinogenic effects.

This study was performed on order to evaluate the amount of the main phytochemicals (vitamin C, total polyphenols, and total flavonoids) content and their antioxidant activity.

**Results:**

The results obtained revealed that the average amounts of vitamin C within the studied genotypes were: 360.22 mg/100 g frozen pulp (var. *transitoria* f. *ramosissima*, altitude 1250 m) and 112.20 mg/100 g frozen pulp (var. *assiensis*, altitude 440 m), giving a good correlation between the vitamin C content of the rosehip and the altitude. The total polyphenols content varied from 575 mg/100 g frozen pulp (var. *transitoria* f. *ramosissima*) to 326 mg/100 g frozen pulp (var. *lutetiana* f. *fallens*). The total flavonoids content showed the highest value for var. *assiensis* variant 163.3 mg/100 g frozen pulp and the lowest value attributed to var. *transitoria* f. *montivaga* 101.3 mg/100 g frozen pulp. The antioxidant activity of eight rose hip extracts from wild Transylvania populations was investigated through DPPH method. The antioxidant activity revealed a good correlation only with vitamin C content and total polyphenols.

**Conclusion:**

Eight Rose hip fruit species were compared taking into consideration the ascorbic acid, total polyphenols, total flavonoids contents and their antioxidant activity. Based on these results, two of the rosehip genotypes that were analysed could be of perspective for these species’ amelioration, due to their content of phytochemicals mentioned above. These varieties are var. *transitoria* f. *ramosissima* (Bistrita-Nasaud, Agiesel) and var. *transitoria* f. *montivaga* (Bistrita-Nasaud, Salva) which can be used as a potential source of natural antioxidants.

## Background

The genus Rosa contains over 100 species that are widely distributed mostly in Europe, Asia, the Middle East and North America [1]. *Rosa canina L.* (dog rose) is an erect shrub of up to 3.5 meters height, sometimes climbing; its branches are often curved or arched. Petals are white to pale pink, rarely deep pink and fruit ripens late [2]. Information about the biology and biochemistry of *Rosa canina L.* has allowed a good documentation by presenting it in several ways (botanical, agrotechnical, chemical composition and uses).

The *Rosa canina L.* fruits have constituted an important source of food and medicine for many cultures. Common food preparations using rose hips include juice, wine, tea, jelly, jam, as well as mixed with dried salmon eggs [3].

The dog rose hips (*Cynosbati fructus*) comprise several biologically active compounds, such as: sugars, organic acids, pectins, flavonoids, tannins, carotenoids, fatty acids, vitamins (particularly vitamin C and also vitamins B1, B2, K, PP, E), macro- and microelements etc. [4,5]. The nutrients and technological properties were determined in *Rosa canina L.* fruits (rosehips) in order to investigate potential uses [4]. In the scientific literature, the vitamin C content in rose hips is reported to far exceed the one found in citrus fruits [4,6]. Rose hips are known to have the highest vitamin C content (30–1300 mg/100 g) among fruits and vegetables [7]. In addition, rose hips contain other vitamins and minerals, carotenoids, tocopherols, flavonoids, fruit acids, tannins, pectin, sugars, organic acids, amino acids and essential oils [8,9]. Indigenous traditional knowledge and western science have revealed its potential for significant nutritional and therapeutic benefits among natural antioxidants [10].

*Rosa canina L.* is well-known for its high phenolic contents. These compounds are known to have antioxidant, antimutagenic and anticarcinogenic effects. Polyphenol compounds are potential antioxidant substances and protective agents against the development of human disease [11,12].

Recent studies revealed that the *Rosa canina L.* extracts were effective on growth inhibition and biofilm formation in methicillin-resistant *Staphylococcus aures* (MRSA) [13,14].

Its seeds are rich in oil and mineral substances. The fatty acids from the dog rose oil are mainly: the linoleic, oleic, linolenic, palmitic, stearic and arachidonic acid [15].

Fruits (hips) have long been used in the traditional prevention and therapy of common cold and other infections, as a diuretic agent and for the treatment of various inflammatory diseases. So far, none of these indications of clinical effectiveness have been proved except for osteoarthritis [16-18]. Citing recent results of other authors’ research, Kiliçgun and Dehen [19] stated that the hips display an anti-inflammatory, antioxidant and anti-mutagen effect. Indigenous traditional knowledge and western science have revealed the potential of nutritional and therapeutic benefits among natural antioxidants [10]. The substances within the dog rose fruit (hips) are endowed with vitaminisant, astringent, colagogue, choleretic, diuretic, antidiarrhoea, antioxidant properties, etc. [20]. Moreover, the research done by Orhan et al. [5], showed that the dog rose hips also have antidiabetic properties (probably due to their monosaccharids, oligosaccharids and pectins content).

Many works dealing with the nutritional value and chemical composition of some rose species fruits, especially *Rosa canina L.,* have been published, but no detailed study concerning the variation of biochemical composition of rosehip based on the biotype or form in different climatic conditions has been done.

National research concerning the biochemical composition of wild rose fruit pointed out the value of biochemical aspect only at the species level and not at the biotypes level. The purpose of this study was to compare the chemical composition of eight forms of *Rosa canina L.* from Transylvania and to estimate the possibility of rose hips fruits’ using in food and food additive sectors.

## Results and discussion

### Ascorbic acid

**Figure 1:**
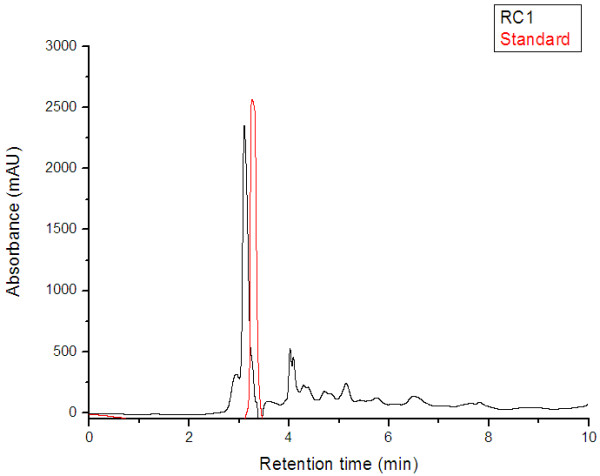
HPLC-UV–vis chromatograms of the standard L- ascorbic acid (t_R_ = 3.2 min) and L-ascorbic acid from frozen pulp rose hips (RC1-var. transitoria f. ramosissima) (t_R_ = 3.1 min).

The HPLC/UV–vis system was used to evaluate the concentration of ascorbic acid in frozen rose hips pulp, with L-ascorbic as standard for the calibration curve (R^2^ = 0.9949). The HPLC chromatograms of the standard L-ascorbic acid and L-ascorbic acid from frozen rose hips pulp of RC1 (var. *transitoria* f. *ramosissima*) are presented in Figure 1.

**Figure 2:**
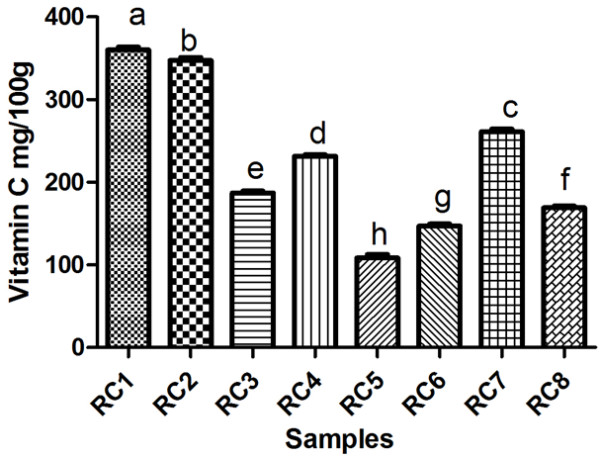
Ascorbic acid content in RC1, RC2, RC3, RC4, RC5, RC6, RC7, RC8 rose hip samples.

The amounts of ascorbic acid in samples (frozen rose hips pulp) are presented in Table 1 and Figure 2. Regarding the content of vitamin C, the results have large variability between the average amounts of vitamin C within the studied genotypes: 360.22 mg/100 g frozen pulp for RC1 (var. *transitoria* f. *ramosissima* Bistrita-Nasaud, Agiesel, altitude 1250 m) followed by RC2 (var. *transitoria* f. *montivaga* Bistrita-Nasaud, altitude 950 m), while the lowest concentration was obtained for RC5 (var. *assiensis* Cluj, altitude 440 m), respectively 112.20 mg/100 g frozen pulp.

**Table 1:** The ascorbic acid concentrations in frozen rose hip pulp

**Sample**	**Variety and form**	**Altitude (m)**	**Ascorbic acid (mg /100 g )**
RC1	var. *transitoria* f. *ramosissima* (Bistrita-Nasaud Agiesel)	1250	360.22 ±2.87 ^a^
RC2	var. *transitoria* f. *montivaga* (Bistrita-Nasaud, Salva)	950	347.50±2.12 ^b^
RC3	var. *andegavensis* f. *transsilvanica* (Salaj, Buciumi)	336	187.20±1.20 ^e^
RC4	var. *andegavensis* f. *vinealis* (Bistrita-Nasaud, Beclean)	530	231.50±1.34 ^d^
RC5	var. *assiensis* (Cluj, Manastur)	440	112.20±2.82 ^h^
RC6	var. *lutetiana* f. *fallens* (Satu-Mare, Petea)	275	147.10±0.07 ^g^
RC7	var. *lutetiana* f. *flexibilis* (Cluj, Feleac);	711	261.30±1.13 ^c^
RC8	var. *lutetiana* f. *psilogyna* (Arad, Galsa)	173	169.10±0.14 ^f^

Values are mean ±SD, n=3; in each column, mean values with different letters are significantly different at p < 0.05.

Identical letters denote the lack of statistically significant differences at p<0.05.

These results are in agreement with the literature data. The concentration of ascorbic acid in different rose species was reported to be of 106–2712 mg/100 g fresh pulp in some studies conducted in different agro-climatic regions of Turkey [4,9,21-26]. Also, Novajan et al. [27] reported that the ascorbic acid amount found in rose hip varied between 211–417.5 mg/100 g. fresh pulp, which is in agreement with our results. Other studies reported higher values of ascorbic acid, for example, Jablanska-Rys et al. [28] (1252 mg/100 g fresh pulp), Rosu et al. [29] (643 mg/100g fresh pulp), or lower amounts of vitamin C between 40–47 mg/100 g fresh pulp [30,31].

The vitamin C content is one of the most important features in the rose hip study areas. Differences in ascorbic acid contents could result from the variations in altitude, species, variety, ecological factors, and harvesting period [23]. The decrease in vitamin C content in plants may also be the result of the environment oxygen level, the amount of light reaching the plants, variations in endogenous plant growth regulators and the temperature. The rose hips frozen fruits have a lower content of vitamin C than fresh fruit - approximately 30.1% when compared with literature data [9].

Our study revealed the increase in vitamin C content with altitude; the highest concentration of ascorbic acid being found in RC1 (360.22 mg/100 g) and RC2 (347.50 mg/100), situated in the same region of Transylvania (Tibles Mountain and Salva) at 1250 m, respectively 950 m (Table 1 and Figure 2). The lowest concentration in vitamin C determined for RC5 (112.20 mg/100), a variety that grows in Cluj-Napoca, could be attributed to the polluted air or soil of this city. Differences are also noticed in the morphological features of RC1, RC2 and RC5 regarding the colour, fruit shape, fruit taste, flesh content, presence or absence of thorns. Some authors [32] assert that the taxonomic assignment level lower than species (i.e. subspecies and variety) plays a great role in what concerns the level of vitamin C in rose hips.

The literature data shows important differences in the content of ascorbic acid in the hips of roses from the *Caninae* section [33]. *Rosa canina L.* usually has low content of ascorbic acid (510 mg/100g) when compared to other studied species, such as *Rosa drumalis, Rosa villosa*[34].

Significant differences were found in the vitamin C content of all the rose hips’ varieties that were analysed (p<0.05).

### Total polyphenol content (TPC)

The polyphenol compounds are important plant-constituents because of their free radical scavenging ability, facilitated by their hydroxyl groups. Total polyphenolic content (TPC) was estimated and expressed using the Folin–Ciocalteu method. Gallic acid was used as standard and the results (as gallic acid equivalent, mg/100 g frozen pulp) were expressed as means ± standard deviation of triplicate analysis.

**Figure 3:**
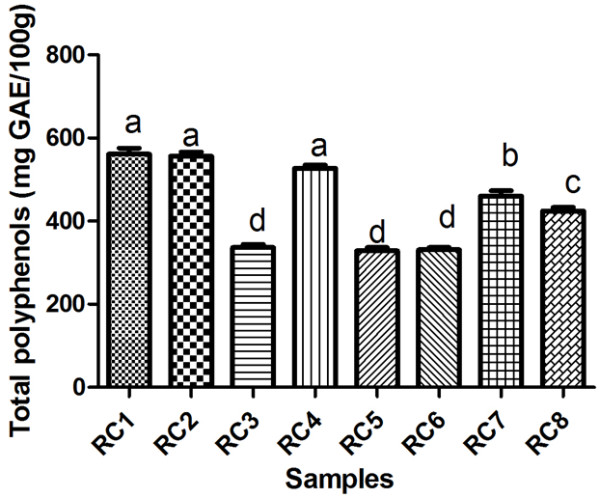
Total polyphenols content in RC1, RC2, RC3, RC4, RC5, RC6, RC7, RC8 rose hip samples.

Table 2 and Figure 3 depict the total polyphenols content of the extracts that were analysed. The total polyphenols content of the *Rosa canina L.* hips extracts registered values between: 575.0 mg GAE/100 g for RC1 (var. *transitoria* f. *ramosissima* from Bistrita-Nasaud, Agiesel) and 326.5 mg GAE/100 g for RC6 (var. *lutetiana* f. *fallens* from Satu-Mare, Petea). High concentrations of total phenolics were also found in the extracts of RC2 (var. *transitoria* f. *montivaga* (Bistrita-Nasaud, Salva) 548.0 mg GAE/100 g, followed by RC4 (var. *andegavensis* f. *vinealis* (Bistrita-Nasaud, Beclean) 534.5 mg GAE/100 g.

**Table 2:** Total polyphenols content in frozen rose hip pulp

**Sample**	**Variety and form**	**mg GAE/100 g frozen pulp**
RC1	var. *transitoria* f. *ramosissima* (Bistrita-Nasaud Agiesel)	575.1±14.64^a^
RC2	var. *transitoria* f. *montivaga* (Bistrita-Nasaud, Salva)	548.3±11.31^a^
RC3	var. *andegavensis* f. *transsilvanica* (Salaj, Buciumi)	343.4±12.02^d^
RC4	var. *andegavensis* f. *vinealis* (Bistrita-Nasaud, Beclean)	534.7±18.50^a^
RC5	var. *assiensis* (Cluj, Manastur)	335.5±11.31^d^
RC6	var. *lutetiana* f. *fallens* (Satu-Mare, Petea)	326.3±5.65^d^
RC7	var. *lutetiana* f. *flexibilis* (Cluj, Feleac);	472.6±13.40^b^
RC8	var. *lutetiana* f. *psilogyna* (Arad, Galsa)	424.8±9.89^c^

Values are mean ±SD, n=3; in each column, mean values with different letters are significantly different at p < 0.05.

Identical letters denote the lack of statistically significant differences at p<0.05.

These data are in agreement with some literature data, such as: Nowak et al. [35], who obtained 990 mgGAE/100g dry plant, Fattahi et al. [36] with an average of 199 mg GAE/100g and Yoo et al. [37], who reported 818 mg GAE/100g fresh fruit. Duda-Chodak et al. [38], who studied the content of the main bioactive compounds in 15 herbal products used for their antioxidant activity, like oak bark, elder fruits, dog rose, reported for *Rosa canina L.* the lowest concentration in total polyphenols, an average of 110 mg/100g dry fruits. Same results were obtained by Yilmaz et al. [39] for rose hip 102 mg/100 g dry fruits, Coruh et al. [40], who reported 78.13 mg GAE/100 g dry weights, data which are lower than in our study. Ercisli [9] reported an average that is much higher than ours in what concerns the polyphenols in rose hip, about 9600 mg/100 g dry fruit.

According to these results, the total polyphenols of *Rosa canina L.* are higher than blueberry (2.7-3.5 mg/g), black currant (3–4 mg/g) or raspberry (2.7-3.0 mg/g), reported by Heinonen, Meyer and Frankel [41]. The content of the phenolic shows that the rose hips could be considered a very good source of this compound compared to other fruit species.

The differences among the rose species regarding the phenolics compounds could be due to genetic derivation, because all plants were harvested in the same period (september-october), but in different ecological conditions. It was reported that the plant genotype, cultivation site and technique affect the total phenolic content in fruit [42].

### Total flavonoids

The isolation of flavonoids from biological samples is important for the quantitative analysis. Because of the complexity of the biological matrix, other components could interfere in the analysis. The most common procedure used to evaluate the total flavonoid content is a spectrophotometric assay, based on the formation of a complex between the aluminium ion and the carbonyl and hydroxyl groups of the flavonoids. Some works have demonstrated that this procedure has different responses depending on the flavonoid structure [43]. Our results are expressed as mg. quercitin/100g frozen pulp.

**Figure 4:**
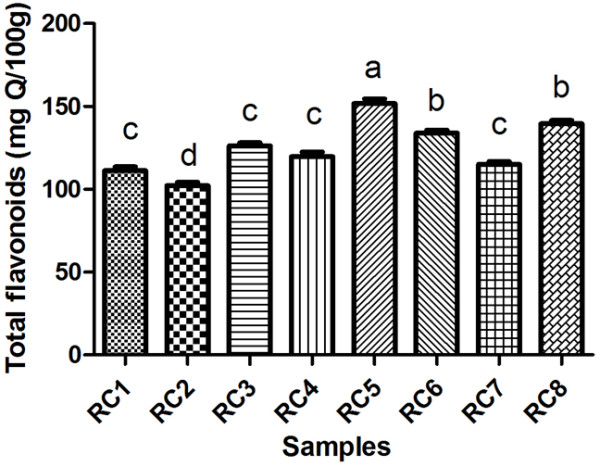
Total flavonoids content in RC1, RC2, RC3, RC4, RC5, RC6, RC7, RC8 rose hip samples.

The results regarding the total flavonoid content are depicted in Table 3 and Figure 4; significant differences can be observed in the total flavonoids content for all the rose hips varieties that were analysed (p<0.05).

**Table 3:** Total flavonoids content in frozen rose hip pulp

**Sample**	**Variety and form**	**mg QE/100 g frozen pulp**
RC1	var. *transitoria* f. *ramosissima* (Bistrita-Nasaud Agiesel)	110.2±3.41^f^
RC2	var. *transitoria* f. *montivaga* (Bistrita-Nasaud, Salva)	101.3±3.33^g^
RC3	var. *andegavensis* f. *transsilvanica* (Salaj, Buciumi)	126.5±4.41^c^
RC4	var. *andegavensis* f. *vinealis* (Bistrita-Nasaud, Beclean)	119.6±3.58^d^
RC5	var. *assiensis* (Cluj, Manastur)	163.2±5.45^a^
RC6	var. *lutetiana* f. *fallens* (Satu-Mare, Petea)	137.2±4.83^b^
RC7	var. *lutetiana* f. *flexibilis* (Cluj, Feleac);	114.4±3.65^e^
RC8	var. *lutetiana* f. *psilogyna* (Arad, Galsa)	139.7±4.40^b^

Values are mean ±SD, n=3; in each column, mean values with different letters are significantly different at p < 0.05.

Identical letters denote the lack of statistically significant differences at p<0.05.

The results show variability in the content of flavonoids in the hips of roses from sect. *Caninae* and are in agreement with Montazeri et al. [44], who reported 104 mg QE/100 g fresh weight for methanolic extract. Lower concentration in flavonoids was reported by Fattahi et al. [36] with an average of 2 mg/100 mg rose hip, Adamczak et al. [33] and Ghazghazi et al. [45] about 41 mg QE/100 g dry fruit, respectivelly 0.11-0.41mg RE/ml extract. As for *Rosa canina L.,* Ghazghazi also reported an inverse ratio between the content in ascorbic acid and flavonoid which is in agreement with our results; for RC5, with the lowest concentration of ascorbic acid (112 mg/100 g), the corresponding flavonoids content is the highest (163.2 mg QE/100 g), followed by RC6 with 147 mg/100 g vitamin C and 137.2 mg QE/100 g frozen pulp.

Yoo et al. [37] reported a higher content in flavonoids (400 mg QE/100 g fresh fruit) but in *Rosa rubiginosa*, data which is in concordance with Adamczak et al. [33] who obtained the highest concentration in flavonoids for *Rosa rubiginosa* among other 11 species of Rosa L.

Table 4 illustrates the results for total phenolic and total flavonoid content and the flavonoids/phenolics ratio in the studied rose hips.

**Table 4:** Total polyphenols and flavonoids content in frozen rose hip pulp

**Sample**	**mg GAE/100 g frozen pulp**	**mg QE/100 g frozen pulp**	**Ratio flavonoids/ Phenolics**
RC1	575.1±14.64^a^	110.2±3.41^f^	0.19
RC2	548.3±11.31^a^	101.3±3.33^g^	0.18
RC3	343.4±12.02^d^	126.5±4.41^c^	0.37
RC4	534.7±18.50^a^	119.6±3.58^d^	0.22
RC5	335.5±11.31^d^	163.2±5.45^a^	0.49
RC6	326.3±5.65^d^	137.2±4.83^b^	0.41
RC7	472.6±13.40^b^	114.4±3.65^e^	0.24
RC8	424.8±9.89^c^	139.7±4.40^b^	0.33

Identical letters denote the lack of statistically significant differences at p<0.05.

These data outline the richest phenolic sources – RC1, RC2, RC4, but the total flavonoids have a smaller share of total phenolics in comparison with RC5, with a ratio of 0.49 and RC6 with a ratio of 0.41, which have the lowest contents in total phenols. It could be supposed that this is due to the rich abundance of anthocyanidines in combination with the other flavonoids. The variation of phenolic compounds content depends on fruits development; in red coloured varieties (like RC1, RC2, RC4) it increases during the ripening stage due to the maximal accumulation of anthocyanines and flavonols [33]. The phenolic acids prevail in RC1, RC2, RC4 and this fact could explain the sour astringent taste.

### Antioxidant activity

#### *DPPH-scavenging activity assay*

The radical scavenging activity of the methanolic *Rosa canina L.* extracts was determined from its DPPH radical quenching ability. Some studies showed that antioxidant activity of plants extracts is correlated with total phenolics rather than with individual phenolic compound [25], so the total phenol content was investigated in this study. It is important to state that different phenols develop different activities, depending on their chemical structure (phenolic acids, flavonols, antocyanidins, stilbens) and the capacity for scavenging free radicals from these classes of compounds differs. The antioxidant properties of a single compound within a group can be different, that’s why the same levels of phenolic compound do not necessarily correspond to the same antioxidant responses [45].

**Figure 5:**
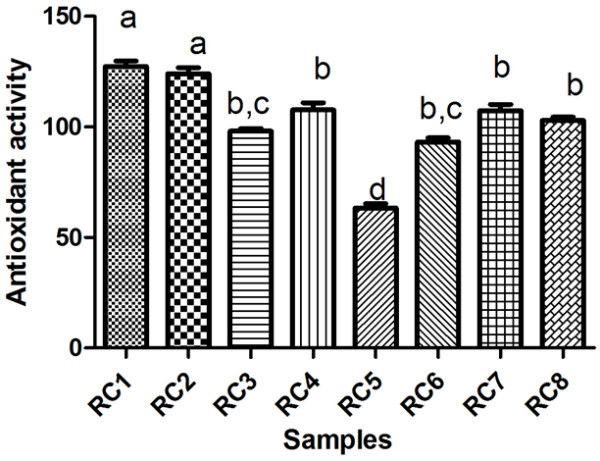
Antioxidant activity expressed as μM Trolox/100 g frozen rose hips.

The results are presented in Table 5 and significant differences between Trolox concentration in all samples analysed (Figure 5) can be observed.

**Table 5:** Antioxidant activity for frozen fruit by DPPH method

**Sample**	**Variety and form**	**Antioxidant activity μM Trolox/100 g pulp**
RC1	var. *transitoria* f. *ramosissima* (Bistrita-Nasaud Agiesel)	127.8 ±1.41^a^
RC2	var. *transitoria* f. *montivaga* (Bistrita-Nasaud, Salva)	124.01±2.40^a^
RC3	var. *andegavensis* f. *transsilvanica* (Salaj, Buciumi)	98.26 ±1.53^bc^
RC4	var. *andegavensis* f. *vinealis* (Bistrita-Nasaud, Beclean)	108.02 ±2.78^b^
RC5	var. *assiensis* (Cluj, Manastur)	63.35±0.70^d^
RC6	var. *lutetiana* f. *fallens* (Satu-Mare, Petea)	91.82±3.16^bc^
RC7	var. *lutetiana* f. *flexibilis* (Cluj, Feleac);	107.30±2.23^b^
RC8	var. *lutetiana* f. *psilogyna* (Arad, Galsa)	102.27±3.69 ^b^

Values are mean ±SD, n=3; in each column, mean values with different letters are significantly different at p < 0.05.

Identical letters denote the lack of statistically significant differences at p<0.05.

Antioxidant activity of *Rosa canina L.* fruits ranged from 63.35 μM Trolox/100 g sample for RC5 (var. *assiensis*, Cluj, Manastur) to 127.8 μM Trolox/100 g sample for RC1 (var. *transitoria* f. *ramosissima*, Bistrita-Nasaud, Agiesel).

Our results, expressed in μg Trolox/ml, varied between 18.12 μg Trolox/ml for RC5 to 36.6 μg Trolox/ml for RC1, which are in accordance with Wenzig et al. [46] (13.7 μg Trolox/ml – 25 μg Trolox/ml) and Ghazghazi et al. [45] (12.5 μg Trolox/ml - 22.6 μg Trolox/ml).

### Correlations

Phenolic compounds are considered to be a major group of compounds that contribute to the antioxidant activities of botanical materials because of their scavenging ability, made possible by their hydroxyl groups. The antioxidant capacity of phenolic compounds is mainly due to their redox properties, which allow them to act as reducing agents, hydrogen donors, singlet oxygen quenchers or metal chelators. Phenolics are believed to be the major phytochemicals responsible for the antioxidant activity of plant materials [47].

**Figure 6:**
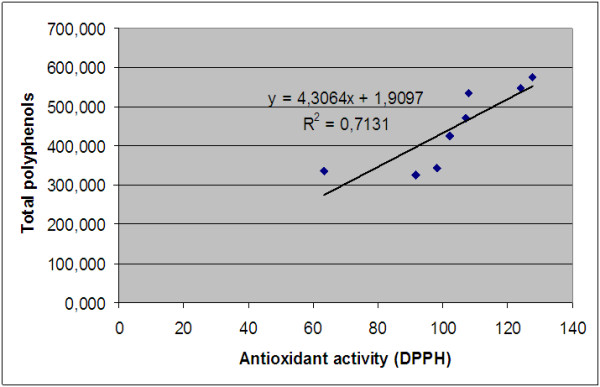
Correlation between the antioxidant activity and the total polyphenols content.

The relation between the polyphenols content and the antioxidant capacity was determined by using linear correlations. There was a good linear correlation (R^2^ = 0.713, p < 0.05) between the total phenols content and the scavenging radical of the rose hip extracts (Figure 6). This result indicated that the radical scavenging capacity of each extract could be related to their concentration of phenolic hydroxyl groups. The antiradical activity of phenolic compounds depends on their molecular structure, on the availability of phenolic hydrogen, on the stabilization possibility of the resulting phenoxyl radicals formed by hydrogen donation, in a variety of extraction solvent polarity and thus different extractability [41].

In previous studies dealing with correlations of the phenolics compounds to DPPH, the scavenging activity showed that the polyphenols were involved with a very different coefficient of determination R^2^ = 0.723 [41]; R^2^ = 0.411 [48]; R^2^ = 0.086 [36].

For other wild fruits, the positive correlations between the total phenols and the antioxidant activity, were recorded by Egea et al. [12], Kilicgun et al. [11], Ghazghazi et al. [46]. There are also reports concerning the lack of correlations between the total phenolic compounds and the radical scavenging [49]. The high free radical scavenging capacity of wild fruits could also be attributed to the presence of other bioactive components, such as vitamin C, tocopherols, pigments, as well as the interaction of these compounds [50].

**Figure 7:**
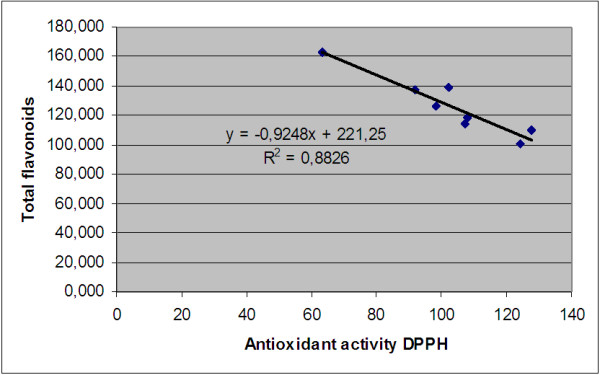
Correlation between the antioxidant activity and the total flavonoids content.

In our study, flavonoids are negatively correlated with scavenging radical of the extracts as it can be observed in Figure 7. This lack of a relationship is in agreement with other reports [48] that also obtained negative correlation. In other studies, a low correlation was obtained [45,51,52], pointing out that flavonoids did not contribute to the antioxidant activity of some fruits.

**Figure 8:**
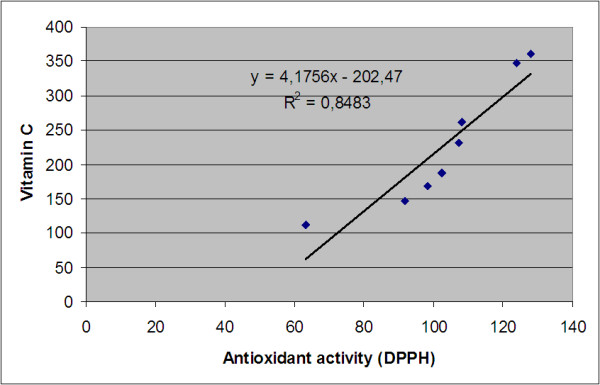
Correlation between antioxidant activity and ascorbic acid content.

Regarding the ascorbic acid and the radical scavenging capacity, our study reveals a good correlation (R^2^ = 0.848) which is presented in Figure 8, being in accordance with Samee et al. [53] (R^2^ = 0.7725).

**Figure 9:**
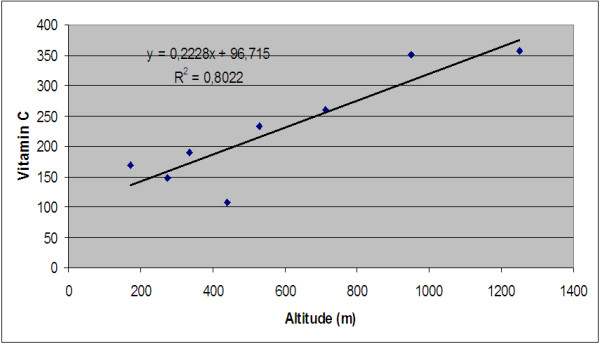
Correlation between altitude and ascorbic acid content.

The correlation between the ascorbic acid and the altitude (Figure 9) with R^2^ = 0.802, suggests that the content in vitamin C increases with altitude. Due to the decrease in the oxygen content at the high altitude, plants get rid of oxidative stress and the vitamin C contents increases. The respiratory rate of fruit is also reduced at low temperature and at high altitude. As a result, the decrease in oxygen concentration in fruit prevents the degradation of the ascorbic acid in plants [54]. Therefore, the decrease in the oxygen concentration prevents the degradation of vitamin C in plants. Biosynthesis and the accumulation of vitamin C in fruits of rosehip are significantly influenced by altitude.

## Conclusions

The results obtained throughout this study show large variability in the content of the active compounds of the *Rosa canina L.* biotypes. The differences in the amount of vitamin C, in the total polyphenols and flavonoids content, were significant. The level of vitamin C content in the rosehip fruit varies according to biotype and altitude, depending on the geographic area of their origin, with higher values at high altitudes. Same degree of variability was observed both individually as well as between biotypes collected from the same geographical area or from various areas. The results obtained show that there are significant differences in the TPC values of the *Rosa canina L.* varieties collected on different locations throughout Transylvania. The total polyphenol content (TPC) and the antioxidant activity are both parameters of quality for rosehips, regarding its biological properties, and both assays should be applied for the quality control of rosehip fresh, frozen or dried. In this study, a high correlation was proved between the total polyphenols, the ascorbic acid content and the antioxidant scavenging capacity.

The antioxidant activity of the rose hip extract could be predicted by the total phenolics and ascorbic acid which suggests that these fruits acted as antioxidant by multiple components.

The conclusion drawn from this study was the fact that two varieties could be selected from the eight biotypes, considered as precious forms in terms of its high content in biologically active substances: these are the var. *transitoria* f. *ramosissima* (Bistrita-Nasaud, Agiesel), altitude 1120 m and the var. *transitoria* f. *montivaga* (Bistrita-Nasaud, Salva), altitude 950 m.

## Experimental

### Plant material

A total of eight *Rosa canina L.* varieties were selected from: Cluj-Napoca, Satu-Mare, Bistrita-Nasaud, Salaj and Arad regions. The samples were collected from different areas and altitudes (ranged between 275–1250 m). These areas’ annual average temperature is somewhere between 8.7°C and 10.8°C, specific for mountain vegetation or plain vegetation. However, several factors including genotype, climate, region, harvesting time and altitude, may be responsible for various chemical composition and consequently, activities [45].

From September to October 2011, rose hips of *Rosa canina L.* were collected from spontaneous flora of Transylvania. Plants were taxonomically identified and the form and variety of each biotype to be analysed, was determinate after a preliminary botanical analysis.

1. var. *transitoria* f. *ramosissima* (Bistrita-Nasaud, Agiesel);

2. var. *transitoria* f. *montivaga* (Bistrita-Nasaud, Salva);

3. var. *andegavensis* f. *transsilvanica* (Salaj, Buciumi);

4. var. *andegavensis* f. *vinealis* (Bistrita-Nasaud, Beclean);

5. var. *assiensis* (Cluj, Manastur);

6. var. *lutetiana* f. *fallens* (Satu-Mare, Petea);

7. var. *lutetiana* f. *flexibilis* (Cluj, Feleac);

8. var. *lutetiana* f. *psilogyna* (Arad, Galsa).

Rose hips without calyxes were washed several times with water and kept in a freezer at −20°C. Whole rose hips were used for biochemical determination (content of vitamin C, polyphenols, flavonoids and antioxidant activity assays).

### Chemicals

Methanol, L-ascorbic acid standard 99%, were purchased from Sigma Chemical Co. (Madrid, Spain). All others chemicals were purchased from Merck (Darmstadt, Germany).

### Vitamin C extraction and determination

#### *Sample preparation*

For the ascorbic acid extraction, 0.5 grams of frozen rose hips, in three replicates each, was homogenised by grinding the sample one minute with 2.5 ml 3% H_3_PO_4_ and 8% acetic acid in aqueous solution, followed by 10 minute centrifugation at 3000 rpm and the supernatant was filtered through a 0.45 μm filter and kept at 4°C, following the procedure described by Hernandez et al. [55] with some modifications.

#### *HPLC separation and identification of ascorbic acid*

The extract obtained was diluted 2 times in double distilled water and injected into the HPLC system. The HPLC analyses were carried out on the Agilent 1200 system with a UV–vis detector, using a reverse phase Eclipse XDB-C18 column (150 × 4.6 mm), 5 μm. An isocratic mobile phase was used: water/acetonitril/formic acid 94/5/1 (v/v/v) with a flow rate of 0.5 ml/minute. All chromatograms were monitored at 240 nm. The HPLC peaks were assigned by spiking the samples with standard ascorbic acid (purity 99%) and comparing the UV spectra and retention time (ascorbic acid 3.2 min). The regression coefficient R^2^ of the calibration curve for ascorbic acid (Y= 52356x - 2432.8) was 0.9949. Calibration curve was obtained from 5 concentrations of external standard (0.2-0.8 mg/ml). The limits of quantification (LOQ) and detection (LOD) of ascorbic acid were 0.19 mg/ml and 0.057 mg/ml.

The results were expressed as: milligram ascorbic acid/100 g frozen rose hip.

#### *Polyphenols extraction and determination*

The total phenolics were determined by using the Folin-Ciocalteu method of Singleton et al. [56].

#### *Polyphenols extraction*

About 2 grams of frozen rose hip, in three replicate, was extracted by grinding the sample with 10 ml of acidified methanol (90:10 v/v, MeOH/HCl 37%). The homogenate was sonicated for 30 minutes, centrifuged at 3500 rpm for a period of 10 minute. The extract was separated and the residual tissue was re-extracted three times. The filtrates were combined in a total volume extract, filtered, dried using a rotary evaporator set at 40°C, dissolved in methanol and kept at 4°C for further analyses.

#### *Total polyphenols quantification (TPC)*

The methanolic extracts (1ml) were added to a 100ml volumetric flask; 60 ml water and 5 ml Folin-Ciocalteu reagent were added and mixed. After 5 min., 15 ml of 7.5% Na_2_CO_3_ solution was added with the mix. The solution was diluted to the volume of 100 ml with ddH_2_O and then allowed to stand for 90 min. in the dark. The absorbance was measured on a multidetection BIOTEK spectrophotometer at 750 nm. The total polyphenols content was calculated using a calibration curve, expressed as mg gallic acid/ ml extract (R^2^ = 0.994) and then reported to 100 g frozen material.

#### *Flavonoids extraction and determination*

The total flavonoids content was measured by the AlCl_3_ colorimetric assay according to Zhishen et al. [57].

#### *Flavonoids extraction*

5 grams of frozen rose hip, in three replicate, was extracted by grinding the sample with 10 ml of acidified methanol (90:10 v/v, MeOH/HCl 37%). The homogenate was sonicated for 30 minutes, centrifuged at 3500 rpm for a period of 10 minute. The extract was separated and the residual tissue was re-extracted three times. The filtrates were combined in a total extract, separated, filtered and purified on an Amberlite XAD-4 column (20 cm height/2 cm diameter/ 20 grams resin) with acidified ddH_2_O (pH = 2) and methanol. A volume of 2 ml sample was washed with 100 ml acidified water (pH = 2), then with 300 ml distilled water for carbohydrates removal and finally with 300 ml methanol. The purified extracts were evaporated using a rotary evaporator set at 35°C to a volume of 3.5 ml.

#### *Flavonoids determination*

An aliquote (0.1 ml) of extracts was added to 10ml volumetric flask containing 4 ml ddH_2_O. Then, 0.3 ml 5% NaNO_2_ solution was added. After 5 minutes 0.3 ml 10% AlCl_3_ solution was poured into the flask and maintained for another 6 minutes, after which 2 ml 1M NaOH solution was added. The total volume was completed up to 10ml with ddH_2_O. The solution was mixed and the absorption was measured at 510 nm. The total flavonoids content was calculated using a calibration curve, expressed as mg quercetin/ml extract (R^2^ = 0.986) and then reported to 100 g frozen material. Samples were analysed in triplicates.

#### *Antioxidant activity determination (DPPH)*

The DPPH scavenging activity assay was registered according to the method reported by Brand-Williams et al. [58]. 80 μM of DPPH solution was freshly prepared in 98% methanol and sonicated for 15 min. 2800 μl of DPPH solution was allowed to react with 400 μl sample and the absorption was measured at 515 nm, for 60 min on a multidetection BIOTEK spectrophotometer. The chemical kinetics of the extracts were recorded and the antioxidant capacity was calculated using a calibration curve, expressed in μM Trolox/L (R^2^ = 0.999).

The antioxidant activity was calculated as follows:

$$\%\;\mathrm{DPPH}\;\mathrm{scavenging}\;\mathrm{activity}\;=\;\left(1-\left[A_{\mathrm{sample}}/A_{\mathrm{control}\;t=0}\right]\right)\cdot 100$$

The results are then reported as μM Trolox equivalent/100 g frozen sample.

### Statistical analysis

Experimental results were expressed as mean ± standard deviation (SD). All measurements were replicated three times and the data were analysed using analysis of variance. In order to determine the significant differences between values, an analysis of variance (ANOVA) and a Newman-Keuls multiple Comparative Test were performed. Significance of difference was defined at the 5% level (p < 0.05). All statistical analysis was carried out using the Graph Pad Version 4.0 (Graph Pad Software Inc; San Diego, CA, USA).

## Abbreviations

TPC: Total polyphenols content; DPPH: Radical scavenging activity of antioxidants against free radicals like the 1.1-diphenyl-2-picrylhydrazyl; HPLC: High performance liquid chromatography; GAE: Gallic acid equivalent; QE: Quercetin equivalent; RC1: *Rosa canina L.* sample no.1; var: Variety; f: Form.

## Competing interests

The authors declare that they have no competing interests.

## Authors’ contributions

All authors contributed equally to the extraction procedures, polyphenols, flavonoids, ascorbic acid analysis and preparation of the paper, samples collection, statistical analysis and preparation of the paper. All authors read and approved the final form of the manuscript.

## References

[B1] Nilsson O Davis PH Rosa Flora of Turkey and the East Aegean Islands 1997 4 Edinburgh: Edinburgh University Press 106 128

[B2] Ercisli S Rose (*Rosa* spp.) germplasm resources of Turkey Genet Resour Crop Eval 2005 52 787 795 10.1007/s10722-003-3467-8

[B3] Moerman DE Native American ethnobotany 2002 Portland, OR: Timber Press 482 486

[B4] Demir F Özcan M Chemical and technological properties of rose (*Rosa canina* L.) fruits grown wild in Turkey J Food Eng 2001 47 333 336 10.1016/S0260-8774(00)00129-1

[B5] Orhan N Aslan M Hosbas S Deliorman O Antidiabetic effect and antioxidant potential of *Rosa canina* fruits Phcog Mag 2009 5 309 315 10.4103/0973-1296.58151

[B6] Ercisli S Esitken A Fruit characteristics of native rose hip (Rosa spp.) selections from the Erzurum province of Turkey NZ J Crop Hortic Sci 2004 32 51 53 10.1080/01140671.2004.9514279

[B7] Ziegler SJ Meier B Sticker O Fast and selective assay of L-ascorbic in rose hips by RP-HPLC coupled with electrochemical and/or spectrophotometric detection Planta Med 1986 5 383 387 17345347 10.1055/s-2007-969192

[B8] Chai JT Ding ZH Nutrients composition of Rosa laevigata fruits Sci Technol Food Ind 1995 3 26 29

[B9] Ercisli S Chemical composition of fruits in some rose (Rosa spp.) species Food Chem 2007 104 1379 1384 10.1016/j.foodchem.2007.01.053

[B10] Yi O Jovel EM Towers GHN Wahbe TR Cho D Antioxidant and antimicrobial activities of native Rosa sp. from British Columbia, Canada Int J Food Sci Nutr 2007 58 3 178 189 10.1080/09637480601121318 17514536

[B11] Kilicgun H Altiner D Correlation between antioxidant effect mechanisms and polyphenol content of Rosa canina Phcog Mag 2010 6 23 238 241 10.4103/0973-1296.66943 20931086 PMC2950389

[B12] Egea I Sanchez-Bel P Romajaro F Pretel MT Replace synthetic additives in functional foods as a natural antioxidant Plants Foods Hum Nutr 2010 65 121 129 10.1007/s11130-010-0159-3 20198440

[B13] Serteser A Kargioglu M Gok V Bagci Y Özcan MM Arslan D Determination of antioxidant effects of some plant species wild growing in Turkey Int J Food Sci Nutr 2008 59 643 651 10.1080/09637480701602530 19382350

[B14] Quave CL Plano LRW Pantuso T Bennet BC Effects of extracts from Italian medicinal plants on plantonic growth, biofilm formation and adherence in MRSA J Ethnopharmacol 2008 118 418 428 10.1016/j.jep.2008.05.005 18556162 PMC2553885

[B15] Ozcan M Nutrient composition of rose (*Rosa canina* L.) seed and oils J Med Food 2002 5 3 137 140 10.1089/10966200260398161 12495585

[B16] Chrubasik C Duke RK Chrubasik S The evidence for clinical efficacy of rose Hip and seed: a systematic review Phytother Res 2006 20 1 3 10.1002/ptr.1729 16395741

[B17] Christensen R Bartels EM Altman RD Astrup A Bliddal H Does the hip powder of *Rosa canina (rosehip) reduce pain in* osteoarthritis patients?-a meta-analysis of randomized controlled trials Osteoarthr Cartilage 2008 16 965 972 10.1016/j.joca.2008.03.001 18407528

[B18] Chrubasik C Wiesner L Black A Müller-Ladner U Chrubasik S A one-year survey on the use of a powder from Rosa canina lito in acute exacerbations of chronic pain Phytother Res 2008 22 1141 1148 10.1002/ptr.2352 18729248

[B19] Kilicgun H Dehen A In vitro antioxidant effect of *Rosa canina* in different antioxidant test systems Phcog Res 2009 1 417 420

[B20] Aresenescu A Pharmacognostical research on the species Rosa canina L 2008 (in Romanian): UMF Cluj-Napoca

[B21] Gunes M Sen SM A study on improvement of wild rose hips (Rosa sp.) growing in Tokat province by selection Horticulture 2001 30 9 16

[B22] Yoruk IH Turker M Kazankaya A Erez ME Batta P Celik F Fatty acid, sugar and vitamin contents in rose hip species Asian J Chem 2008 20 1357 1364

[B23] Dogan A Kazankaya A Fruit properties of rose Hip species grown in lake van basin (eastern Anatolia region) Asian J Plant Sci 2006 5 120 122

[B24] Kazaz S Baydar H Erbas S Variations in chemical composition of Rosa damascena Mill and Rosa canina L fruit Czech J Food Sci 2009 27 178 184

[B25] Wenzig EM Widowitz U Kunert O Chrubasik S Bucar F Knauder E Bauer R Phytochemical composition and in vitro pharmacological activity of two rose hip (*Rosa canina L.)* preparations Phytomed 2008 15 826 835 10.1016/j.phymed.2008.06.012 18707854

[B26] Widen C Ekholm A Coleman MD Renvert S Rumpunen K Erythrocyte antioxidant protection of rose hips (Rosa spp.) Oxi Med Cell Longev 2012 article ID621579 8 10.1155/2012/621579 PMC3399354 22829958

[B27] Novajan S Khalilian F Kiaie FM Atyeh R Arabanian A Chalavi S Extraction and quantitative determination of ascorbic acid during different maturity stages of Rosa canina L fruits J Food Comp Anal 2008 21 300 305 10.1016/j.jfca.2007.11.007

[B28] Jablonska-Rys E Zalewska-Korona M Kalbrczyk J Antioxidant capacity, ascorbic acid and phenolics in wild edible fruits J Fruit Ornam Plant Res 2009 17 2 115 120

[B29] Rosu CM Manzu C Olteanu Z Oprica L Oprea A Ciornea E Zamfirache MM Several fruit characteristics of Rosa spp. Genotypes from Northeastern Region of Romania Not Bot Horti Agrobo 2011 39 2 203 208

[B30] Tayefi-Nasrabadi H Sadigh-Eteghad S Aghdam Z The effects of the hydroalcohol extract of *Rosa canina* L fruit on experimentally nephrolithiasic wistar rats Phytother Res 2012 25 78 85 21544885 10.1002/ptr.3519

[B31] Turkben C Uylaser V Incedayi B Celikkol I Effects of different maturity period and processes on nutritional components of rose hip (Rosa canina L) J Food Agric Environ 2010 8 1 26 30

[B32] Krzaczek W Krzaczek T Phenolic acids of native species of the Rosa L. genus in Poland Acta Soc Bot Pol 1979 48 2 327 336

[B33] Adamczak A Buchwald W Zielinski J Mielcarek S Flavonoid and organic acid content in rose hips (*Rosa L*.,sect.*Caninae* DC.EM.Christ.) Acta Biol Cracov Bot 2012 54 1 1 8

[B34] Gao X Bjork L Trajkovski V Uggla M Evaluation of antioxidant activities of rose hip ethanol extracts in different test systems J Sci Food Agric 2000 80 2012 2027 10.1002/1097-0010(200011)80:14<2012::AID-JSFA738>3.0.CO;2-X

[B35] Nowak R Gawlik-Dziki U Polyphenols of Rosa L. leaves extracts and their radical scavenging activity Z Naturforsch 2007 62c 32 38 10.1515/znc-2007-1-206 17425102

[B36] Fattahi S Jamei R Hosseini Sarghein S Antioxidant and antiradicalic activity of Rosa canina and Rosa pimpinellifolia fruits from West Azerbaijan Iranian J Plant Physiol 2012 2 4 523 529

[B37] Yoo KM Lee CH Lee H Moon B Lee CY Relative antioxidant and cytoprotective activities of common herbs Food Chem 2008 106 929 936 10.1016/j.foodchem.2007.07.006

[B38] Duda-Chodak A Tarko T Rus M Antioxidant activity and total polyphenol content of selected herbal medicinal products used in Poland Herba Polonica 2011 57 1 48 61

[B39] Yilmaz SO Ercisli S Antibacterial and antioxidant activity of fruits of some rose species from Turkey Rom Biotech Lett 2011 16 4 6407 6411

[B40] Coruh S Ercisli S Interactions between galling insects and plant total phenolic contents in Rosa canina L. genotypes Sci Res Essays 2010 5 14 1935 1937

[B41] Heinonen M Meyer A Frankel E Antioxidant activity of berry phenolics on human low density protein and liposome oxidation J Agric Food Chem 1998 46 4107 4112 10.1021/jf980181c

[B42] Scalzo J Politi A Pellegrini N Mezzeti B Battino M Plant genotype affects total antioxidant capacity and phenolics contents in fruit Nutrition 2005 21 207 213 10.1016/j.nut.2004.03.025 15723750

[B43] Popova M Bankova V Butovska D Petkov V Nikolova B Sabatini AG Marcazzan GL Bogdanov S Validated methods for the quantification of biologically active constituents of poplar-type propolis Phytochem Anal 2004 15 235 240 10.1002/pca.777 15311843

[B44] Montazeri N Baher E Mirzajani F Barami Z Yousefian S Phytochemical contents and biological activities of Rosa Canina fruit from Iran J Med Plant Res 2011 5 18 4584 4589

[B45] Ghazghazi H Miguel MG Hasnaoui B Sebei H Ksontini M Figueiredo AC Pedro LG Barroso JG Phenols, essential oils and carotenoids of *Rosa canina* from Tunisia and their antioxidant activities Afr J Biotechnol 2010 9 18 2709 2716

[B46] Prior RL Cao G Martin A Sofic E McEwan J O’Brien C Antioxidant capacity influenced by total phenolic and antocyanin content, maturity and variety of vaccinum species J Agric Food Chem 1998 46 2686 2693 10.1021/jf980145d

[B47] Balasundram N Sundram K Sammar S Phenolic compounds in plants and agr-industrial by-products: Antioxidant Activity. Occurrence and Potential Uses Food Chem 2006 99 1 191 203 10.1016/j.foodchem.2005.07.042

[B48] Turker G Kizilkaya B Cevik N Gonuz A Free radical scavenging activity and phenolic content of edible wild fruits from Kazdagi(Ida Mountain), Turkey J Med Plants Res 2012 6 36 4989 4994

[B49] Yu L Haley S Perret J Harris M Wilson J Qian M Free radical scavenging properties of wheat extracts J Agric Food Chem 2002 50 1619 1624 10.1021/jf010964p 11879046

[B50] Barros L Carvalho AM Morais JS Ferreira ICFR Strawberry-tree, blackthorn and rose fruits: Detailed characterisation in nutrients and phytochemicals with antioxidant properties Food Chem 2010 120 247 254 10.1016/j.foodchem.2009.10.016

[B51] Anagnostopoulou MA Kefalas P Papageorgiou VP Assimopoulou AN Boskou D Radical scavenging activity of various extracts and fractions of sweet orange peel Food Chem 2006 94 19 25 10.1016/j.foodchem.2004.09.047

[B52] Nickvar B Kamalinejad M Izadpanah H In vitro free radical scavenging activity of five salvia species Pak J Pharm Sci 2007 20 291 294 17604251

[B53] Samee W Engkalohakul M Nebbua N Direkrojanavuti P Kamkaen N Correlation analysis between total acid, total phenolic and ascorbic acid contents in fruit extracts and their antioxidant activity Thai Pharm Health Sci J 2006 1 3 196 203

[B54] Guneş M Dolek U Fruit characteristics of promising native rose hip genotypes grown in Mid-North Anatolia Region of Turkey J Food Agric Environ 2010 8 2 460 463

[B55] Hernandez Y Lobo G Gozalez M Determination of vitamin C in tropical fruit: a comparative evaluation of methods Food Chem 2006 96 654 702 10.1016/j.foodchem.2005.04.012

[B56] Singleton VL Orthofer R Lamuela-Raventos RM Lester P Analysis of total polyphenols and other oxidation substrates and antioxidants by means of Folin-Ciocalteu reagent Method enzymol 1999 299 152 178

[B57] Zhishen J Mengcheng T Jianming W The determination of flavonoids contents in mulberry and their scavenging effects on superoxide radicals Food Chem 1999 64 555 559 10.1016/S0308-8146(98)00102-2

[B58] Brand-Williams W Cuvelier ME Berset C Use of free radical method to evaluate antioxidant activity LWT- Food Sci Technol 1995 28 25 30 10.1016/S0023-6438(95)80008-5

